# Heat and Mass Transfer on MHD Flow of a Viscoelastic Fluid through Porous Media over a Shrinking Sheet

**DOI:** 10.1155/2014/572162

**Published:** 2014-09-30

**Authors:** D. Bhukta, G. C. Dash, S. R. Mishra

**Affiliations:** Department of Mathematics, Institute of Technical Education and Research, Siksha “O” Anusandhan University, Khandagiri, Bhubaneswar, Odisha 751030, India

## Abstract

An attempt has been made to study the heat and mass transfer effect in a boundary layer flow through porous medium of an electrically conducting viscoelastic fluid over a shrinking sheet subject to transverse magnetic field in the presence of heat source. Effects of radiation, viscous dissipation, and uniform heat sink on the heat transfer have been considered. The method of solution involves similarity transformation. The coupled nonlinear partial differential equations representing momentum, concentration, and nonhomogenous heat equation are reduced into a set of nonlinear ordinary differential equations. The transformed equations are solved by applying Kummer's function. The exact solution of temperature field is obtained for power-law surface temperature (PST) as well as power-law heat flux (PHF) boundary condition. The interaction of magnetic field is proved to be counterproductive in enhancing velocity and concentration distribution, whereas presence of porous matrix reduces the temperature field at all points.

## 1. Introduction

The fluid flow over a stretching sheet is important in many practical applications such as extrusion of plastic sheets, paper production, glass blowing, metal spinning, polymers in metal spring processes, the continuous casting of metals, drawing plastic films, and spinning of fibers, which all involve some aspects of flow over a stretching sheet or cylindrical fiber [1]. The quality of the final product depends on the rate of heat transfer at the stretching surface. The problem of stretching surface with constant surface temperature was analyzed by Crane [2]. The growing need for chemical reaction and hydrometallurgical industries requires the study of heat and mass transfer with a chemical reaction. There are many transport processes that are governed by the combined action of buoyancy forces due to both thermal and mass diffusion in the presence of chemical reaction effect. These processes are observed in the nuclear reactor safety and combustion systems and solar collectors as well as metallurgical and chemical engineering.

A chemical reaction can be codified as either a homogenous or a heterogeneous process. This depends upon whether it occurs on an interface or as a single-phase volume reaction. A reaction is said to be first order if its rate is directly proportional to the concentration itself.

Chamkha [3] studied the MHD flow of a uniformly stretched vertical permeable surface in the presence of heat generation/absorption and a chemical reaction. The effect of temperature-dependent viscosity on mixed convection flow from vertical plate is investigated by several authors [4, 5]. Ishak et al. [6] investigated theoretically the unsteady mixed convection boundary layer flow and heat transfer due to a stretching vertical surface in a quiescent viscous and incompressible fluid. Mahapatra and Gupta [7, 8] considered the stagnation flow on a stretching sheet. Sammer [9] investigated the heat and mass transfer over an accelerating surface with heat source in presence of magnetic field. Wang [10] studied the stagnation flow towards a shrinking sheet.

Ahmad and Khan [11] investigated boundary layer flow past a stretching plate with suction, heat and mass transfer, and variable conductivity. Elbashbeshy and Bazid [12] studied flow and heat transfer in a porous medium over a stretching surface with internal heat generation and suction/blowing. Cortell [13] also reported the flow and heat transfer of a fluid through porous medium over a stretching surface with internal heat generation. Anjali Devi and Ganga [14] have studied the viscous dissipation effect on nonlinear MHD flow in a porous medium over a stretching porous surface. Several authors [15–17] have also studied the heat transfer problem in different fields. Recently, Midya [18] has investigated the heat transfer in an electrically conducting viscoelastic flow over a shrinking sheet subject to transverse magnetic field.

The objective of the present study is to consider the heat and mass transfer of an electrically conducting viscoelastic fluid flow over a linearly shrinking sheet embedded in a porous medium.

The novelty of the present study is to investigate the effect of porous medium and mass transfer on the flow of a slightly elastic fluid over a shrinking sheet. The results of Midya [18] have been derived as a particular case. The case of stretching sheet cannot be derived as a special case due to mathematical impasse rendering the elasticity of the fluid as a negative quantity.

## 2. Mathematical Formulation

**Figure 1:**
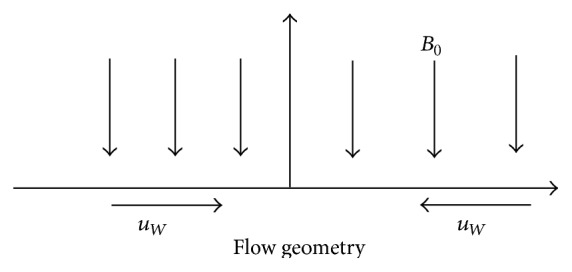


Consider a steady two-dimensional flow of an incompressible electrically conducting second-order viscoelastic fluid over a shrinking surface. In our analysis we have taken *x*-axis along the wall in the direction of motion of the flow, the *y*-axis being normal to it, and *u* and *v* are tangential and normal velocity components, respectively. The applied magnetic field is perpendicular to the plate (see Figure 1). Thus, for the problem under consideration, the equations of momentum, energy, and concentration [19, 20] are given by the following:

(1) $$\begin{array}{c} \frac{\partial u}{\partial x}+\frac{\partial v}{\partial y}=0, \end{array}$$

(2) u∂u∂x+v∂u∂y=υ∂2u∂y2−σB02uρ−υKp′u −k0(∂3u∂x∂y2+v∂3u∂y3+∂u∂x∂2u∂y2−∂u∂y∂2u∂x∂y),

(3) u∂T∂x+v∂T∂y=KρCp∂2T∂y2+μρCp(∂u∂y)2 −1ρCp∂qr∂y+QρCp(T−T∞).

Roseland's approximation for radiation gives *q*_*r*_ = (4*σ* ^*^/3*k*_1_)(∂*T* ^4^/∂*y*). It is assumed that the temperature variation within the flow is such that *T* ^4^ may be expanded in a Taylor's series. Expanding *T* ^4^ about *T*_*∞*_ and neglecting the higher-order terms, we have *T* ^4^ = 4*T*_*∞*_^3^ *T* − 3*T*_*∞*_^4^. Therefore, (3) reduces to the following:

(4) u∂T∂x+v∂T∂y=KρCp∂2T∂y2+μρCp(∂u∂y)2 +16σ∗T∞33k1ρCp∂2T∂y2+QρCp(T−T∞),

(5) $$\begin{array}{c} u\frac{\partial C}{\partial x}+v\frac{\partial C}{\partial y}=D\frac{\partial^{2}C}{\partial y^{2}}-K_{c}\left(C-C_{\infty }\right). \end{array}$$

The boundary conditions are

(6) u=−ax, v=0, T=Tw, C=Cw at  y=0,u⟶0, T⟶T∞, C⟶C∞ as  y⟶∞.

## 3. Solution of the Flow Problem (Methodology)

Equations (1) and (2) admit self-similar solutions of the following form:

(7) $$\begin{array}{c} u=axf^{\prime }\left(\eta \right),\;\;v=-\sqrt{a\upsilon }f\left(\eta \right),\;\;\eta =y\sqrt{\frac{a}{\upsilon }}, \end{array}$$

where *f* is the dimensionless stream function and *η* is the similarity variable. Substituting in (2), we get the following:

(8) f′2−ff′′=f′′′−Rc{2f′f′′′−f′′2−ffiv} −(M+1KP)f′,

where *K*_*p*_ = *K*_*p*_′*a*/*υ* is the permeability parameter, *M* = *σB*_0_^2^/*ρa* is the magnetic parameter, and *R*_*c*_ = *k*_0_ *a*/*υ* is the elastic parameter.

The boundary conditions are

(9) $$\begin{array}{c} f\left(0\right)=0,\;\;f^{\prime }\left(0\right)=-1,\;\;f^{\prime }\left(\infty \right)=0. \end{array}$$

The exact solution (8) with boundary conditions (9) is obtained following Chakrabati and Gupta [21], as follows:

(10) $$\begin{array}{c} f\left(\eta \right)=\frac{e^{-\alpha \eta }-1}{\alpha },\;\alpha =\sqrt{\frac{\left(M+1/K_{p}-1\right)}{\left(1+R_{c}\right)}}. \end{array}$$

## 4. Skin Friction

The shear stress at the wall is defined as

(11) $$\begin{array}{c} \tau_{w}=\mu {\left(\frac{\partial u}{\partial y}\right)}_{y=0}=\mu ax\sqrt{\frac{a}{\upsilon }}f^{\prime \prime }\left(0\right). \end{array}$$

The nondimensional form of skin friction, *C*_*f*_, at the wall is

(12) $$\begin{array}{c} C_{f}=f^{\prime \prime }\left(0\right)=\alpha . \end{array}$$

## 5. Heat Transfer Analysis

Power-law surface temperature (PST) and power-law wall heat flux (PHF) cases are to be considered.

### 5.1. Power-Law Surface Temperature (PST)

In power-law surface temperature, the boundary conditions are given by

(13) $$\begin{array}{c} T_{w}=T_{\infty }+Ax^{2}\;\text{at}\;y=0,\;\;T_{w}=T_{\infty }\;\text{at}\;y⟶\infty . \end{array}$$

Introducing nondimensional quantities *θ*(*η*) = (*T* − *T*_*∞*_)/(*T*_*w*_ − *T*_*∞*_), *P*_*r*_ = *μC*_*p*_/*K*, *E*_*c*_ = *μv* ^2^/*υ*(*T*_*w*_ − *T*_*∞*_) and using (7), the equation (3) becomes

(14) $$\begin{array}{c} \theta^{\prime \prime }+NP_{r}f\theta^{\prime }+NP_{r}\left(\lambda -2f^{\prime }\right)\theta =-E_{c}NP_{r}f^{\prime \prime 2}, \end{array}$$

where *N* = (3*Kk*_1_/4*σ* ^*^ *T*_*∞*_)/(3*Kk*_1_/4*σ* ^*^ *T*_*∞*_ + 4) and *λ* = *Q*/*ρC*_*p*_ *a*.

The boundary conditions (13) become

(15) $$\begin{array}{c} \theta \left(0\right)=1,\;\;\theta \left(\infty \right)=0. \end{array}$$

Substituting the solution for momentum in (14), we get the following:

(16) $$\begin{array}{c} \theta^{\prime \prime }+NP_{r}\frac{e^{-\alpha \eta }-1}{\alpha }\theta^{\prime }+NP_{r}\left(\lambda +2e^{-\alpha \eta }\right)\theta =-E_{c}NP_{r}\alpha^{2}e^{-2\alpha \eta }. \end{array}$$

Introducing the variable *ξ* = *NP*_*r*_ *e* ^−*αη*^/*α* ^2^, (16) is transformed to

(17) $$\begin{array}{c} \xi \frac{d^{2}\theta }{d\xi^{2}}+\left(1+\frac{NP_{r}}{\alpha^{2}}-\xi \right)\frac{d\theta }{d\xi }+\left(\frac{NP_{r}\lambda }{\alpha^{2}}+2\right)\theta =-\frac{E_{c}\alpha^{4}\xi }{NP_{r}} \end{array}$$

with the boundary conditions as follows:

(18) $$\begin{array}{c} \theta \left(\frac{NP_{r}}{\alpha^{2}}\right)=1,\;\;\theta \left(0\right)=0. \end{array}$$

Using confluent hypergeometric function, we get the following:

(19) θ(ξ)=e−αβη[1+EcNPrα24α2+(2+β)NPr] ×Φ(β−2,1+b0,(NPr/α2)e−αη)Φ(β−2,1+b0,(NPr/α2)) −EcNPrα2e−2αη4α2+(2+β)NPr,

where *β* = (*b*_0_ − *a*_0_)/2, *a*_0_ = *NP*_*r*_/*α* ^2^, $b_{0}=\sqrt{{a_{0}}^{2}-4a_{0}\lambda }$.

### 5.2. Power-Law Heat Flux Case (PHF)

The boundary conditions in case of PHF are given by

(20) $$\begin{array}{c} -K\frac{\partial T}{\partial y}=q_{w}=E_{0}x^{2}\;\text{at}\;y=0,\\ T⟶T_{\infty }\;\text{as}\;y⟶\infty , \end{array}$$

where *E*_0_ is a positive constant.

Introducing the similarity variable $T-T_{\infty }=(E_{0}x^{2}/K)\sqrt{\upsilon /a}\psi (\eta )$ and using (7), (4) becomes

(21) $$\begin{array}{c} \psi^{\prime \prime }+NP_{r}f\psi^{\prime }+NP_{r}\left(\lambda -2f^{\prime }\right)\psi =-E_{c}NP_{r}f^{\prime \prime 2}. \end{array}$$

The boundary conditions are

(22) $$\begin{array}{c} \psi =-1\;\text{at}\;\eta =0,\\ \psi ⟶0\;\text{at}\;\eta ⟶\infty . \end{array}$$

Substituting the solution of momentum transport in (21) we get

(23) $$\begin{array}{c} \psi^{\prime \prime }+NP_{r}\frac{e^{-\alpha \eta }-1}{\alpha }\psi^{\prime }+NP_{r}\left(\lambda +2e^{-\alpha \eta }\right)\psi =-E_{c}NP_{r}\alpha^{2}e^{-2\alpha \eta }. \end{array}$$

We introduce a new variable *ξ* = −(*NP*_*r*_/*α* ^2^)*e* ^−*αη*^, and (23) transforms to

(24) $$\begin{array}{c} \xi \frac{d^{2}\psi }{d\xi^{2}}+\left(1+\frac{NP_{r}}{\alpha^{2}}-\xi \right)\frac{d\psi }{d\xi }+\left(\frac{NP_{r}\lambda }{\alpha^{2}\xi }+2\right)\psi =-\frac{E_{c}\alpha^{4}\xi }{NP_{r}} \end{array}$$

with the corresponding boundary conditions as follows:

(25) $$\begin{array}{c} \psi \left(\xi =0\right)=0,\;\;\psi^{\prime }\left(\xi =-\frac{P_{r}}{\alpha^{2}}\right)=-\frac{\alpha }{NP_{r}}. \end{array}$$

The exact solution of (24) subject to the boundary conditions (25) can be written in terms of confluent hypergeometric function in terms of similarity variable *η* and is given by

(26) ψ(η)=(α(1+b0)e−αβη[1+2EcNPrα34α2+(2+β)NPr]  ×Φ(β−2,1+b0,NPrα2e−αη)[1+2EcNPrα34α2+(2+β)NPr]) ×(α2β(1+b0)Φ(β−2,1+b0,NPrα2)   +NPr(β−2)Φ(β−1,2+b0,NPrα2))−1 −EcNPrα2e−2αη4α2+(2+β)NPr.

## 6. Mass Transfer Analysis

The boundary conditions are assigned as

(27) $$\begin{array}{c} -D\frac{\partial C}{\partial y}=m_{w}=E_{1}x^{2}\;\text{at}\;y=0,\\ C⟶C_{\infty }\;\text{at}\;y⟶\infty . \end{array}$$

Introducing the similarity variable $C-C_{\infty }=(E_{1}x^{2}/D)\sqrt{\upsilon /a}\varphi (\eta )$ and using (7) in (5), we get the following:

(28) $$\begin{array}{c} \varphi^{\prime \prime }+S_{c}F\varphi^{\prime }-S_{c}F^{\prime }\varphi -S_{c}K_{c}\varphi =0, \end{array}$$

with the following boundary conditions:

(29) $$\begin{array}{c} \varphi^{\prime }=-1\;\text{at}\;\eta =0,\\ \varphi ⟶0\;\text{at}\;\eta ⟶\infty . \end{array}$$

Again introducing a new variable *ζ* = −(*S*_*c*_/*α* ^2^)*e* ^−*αη*^, (28) becomes

(30) ζd2φd2ζ+[(1−Scα2(α2−M2−1Kp))−ζ]dφdζ  +(2−Kcα2ζ)φ=0.

The corresponding boundary conditions are

(31) $$\begin{array}{c} \varphi \left(\zeta =0\right)=0,\;\;\varphi^{\prime }\left(\zeta =-\frac{S_{c}}{\alpha^{2}}\right)=-\frac{\alpha }{S_{c}}. \end{array}$$

The exact solution of (30) subject to the boundary condition (31) is given by

(32) φ(η)=(e−αηF11(γ−2;1+γ;−Sce−αηα2)) ×((γ−1;  2+γ;−Scα2)(γ−1;  2+γ;−Scα2)αγF11(γ−2;1+γ;−Scα2)   −Scαγ−2(1+γ)F11(γ−1;2+γ;−Scα2))−1,γ=Scα2(α2−M−1Kp).

## 7. Results and Discussion

The present study considers the flow of a viscoelastic incompressible electrically conducting fluid flow past a stretching sheet through a porous medium in the presence of magnetic field, viscous dissipation, and uniform heat source/sink in the presence of chemical reaction. The aim of the following discussion is to bring out the effect of permeability of the medium, plate temperature, and chemical reaction on the flow phenomena.

The heat generation/absorption contributes significantly to nonisothermal heat transfer case. Another consideration of the present study is the saturated porous media. Porous media are very widely used to insulate a heated body to maintain its temperature. They are considered to be useful in diminishing the natural free convection which would otherwise occur intensely on the vertical surface.

Further, the effect of free convection on the flow through porous media plays an important role in agricultural engineering and in petroleum industry in extracting poor petroleum from the crude. Moreover, the present study considers the effect of viscous dissipation which accounts for the heat energy stored in the fluid due to frictional heating.

The following discussion presents the effects of various parameters exhibiting the above phenomena.

**Figure 2:**
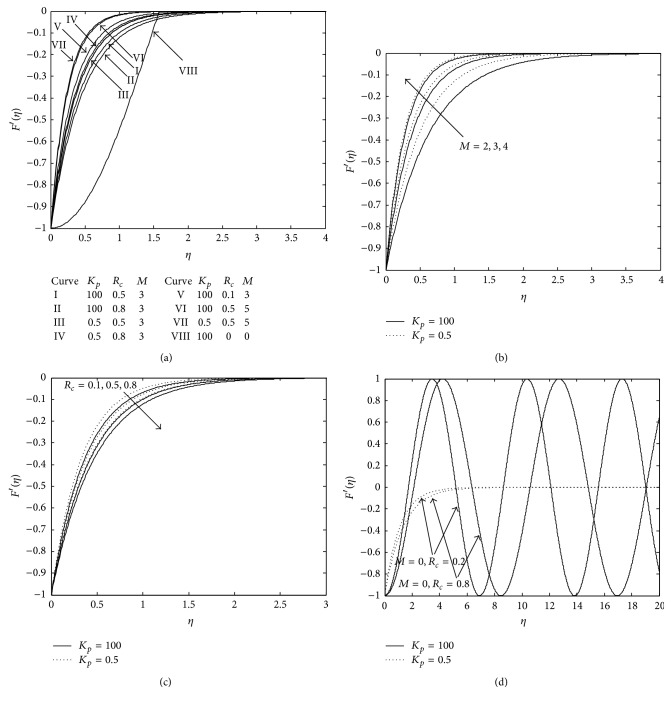
(a) Velocity profile. (b) Velocity profile for *R*_*c*_ = 0.2. (c) Velocity profile for *M* = 3. (d) Velocity profile for *M* = 0.

Figure 2 presents the velocity distribution exhibiting the effect of the porous medium elastic parameter and magnetic parameter. The discussion follows considering the magnitude of velocity profile since all the profiles present the negative values. Curve I and V show that an increase in elastic parameter decreases the velocity in the absence of porous medium (*K*_*p*_ = 100), but the reverse effect is observed in the presence of porous medium (*K*_*p*_ = 0.5, Curves III and IV). This reveals that the presence of porous medium (*K*_*p*_ = 0.5) acts as an insulator to the vertical surface, preventing energy loss due to free convection, as a result of which velocity increases with an increase in elastic parameter. Curves I (*K*_*p*_ = 100) and III (*K*_*p*_ = 0.5) also show that presence of porous medium enhances the velocity. Presence of magnetic field produces Lorentz force which usually resists the motion of the fluid but in the present case it accelerates which is evident from Curves III and VII. This deviation may be attributed to the fact that presence of porous medium and elasticity both contribute to stored energy by preventing energy loss as we know that when viscoelastic fluid flows, a certain amount of energy is stored up in the material as strain energy in addition to viscous dissipation. Therefore, the resistive force due to magnetic field is overcome and hence increase in magnetic parameter enhances the magnitude of the velocity.

Furthermore, it is interesting to note that when *M* = 0 and *K*_*p*_ = 100 (without porous matrix), (10) gives complex value and transitory motion sets in, which is evident from the Figure 1(c).

**Figure 3:**
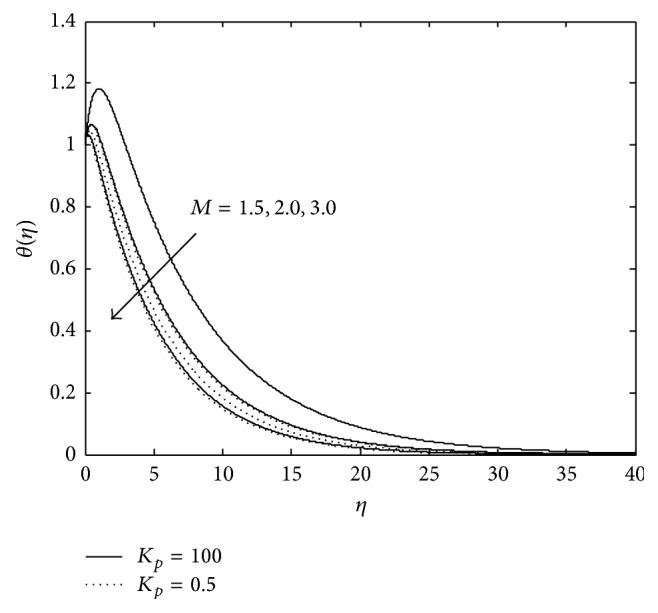
Variation of temperature: *R* = 0.7, *R*_*c*_ = 0.1, *λ* = −0.2, *E*_*c*_ = 0.9, and *P*_*r*_ = 0.9 (PST case).

**Figure 4:**
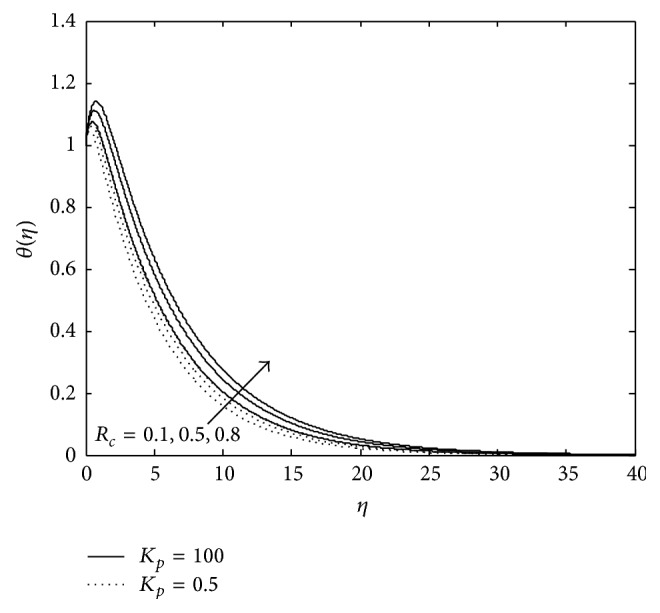
Variation of temperature: *M* = 2, *R* = 1, *P*_*r*_ = 0.9, *E*_*c*_ = 0.6, and *λ* = −0.2 (PST case).

Figures 3 and 4 exhibit the variation of temperature in PST case. It is observed that an increase in magnetic parameter reduces the temperature distribution at all points and the reverse effect is observed in the presence of elastic elements. Moreover, presence of porous matrix (doted curves) reduces the temperature further in both the cases, that is, presence of magnetic field as well as elasticity. An increase in temperature due to the presence of elastic elements may be attributed to the fact that when a viscoelastic fluid is in flow, a certain amount of energy is stored up in the material as strain energy in addition to viscous dissipation but the reduction of temperature in the presence of magnetic field due to resistive Lorentz force which comes into play, as a result some amount of heat energy is dissipated. From Figure 4 it is seen that an increase in *P*_*r*_ leads to a decrease in the temperature in a viscoelastic fluid for a constant value of Eckert number *E*_*c*_ (i.e., in the presence of constant frictional heating stored in the fluid).

Thus, it may be considered that the increase in *P*_*r*_ means slow rate of thermal diffusion. Thus, it may be concluded that thinning of thermal boundary layer thickness is the consequence of fluid with slow rate of thermal diffusion in the presence of magnetic field and porous matrix but the presence of elasticity enhances it.

**Figure 5:**
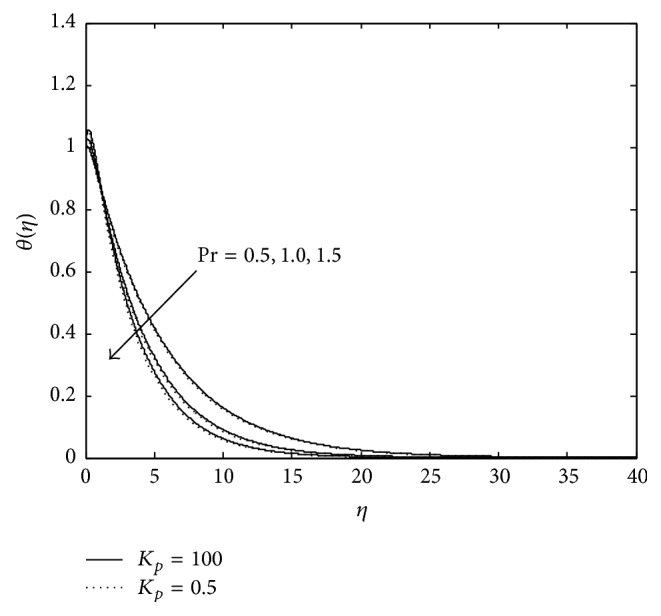
Variation of temperature: *M* = 3, *R*_*c*_ = 0.1, *R* = 0.6, *E*_*c*_ = 1, and *λ* = −0.3 (PST case).

Temperature distribution for different values of *P*_*r*_ in absence/presence of *K*_*p*_ is displayed in Figure 5 taking *R*_*c*_ = 0.1, *M* = 3, *P*_*r*_ = 0.9, *λ* = −0.3, *R* = 0.6, and *E*_*c*_ = 1  as fixed. In the absence of *K*_*p*_ (*K*_*p*_ = 100, bold lines), it is seen that higher Prandtl number fluid causes lower thermal diffusivity and hence reduces the temperature at all points. It is interesting to note that the effect of sink is to lower down the temperature. Similar effect is noticed in the presence of *K*_*p*_ (*K*_*p*_ = 0.5, dotted lines).

**Figure 6:**
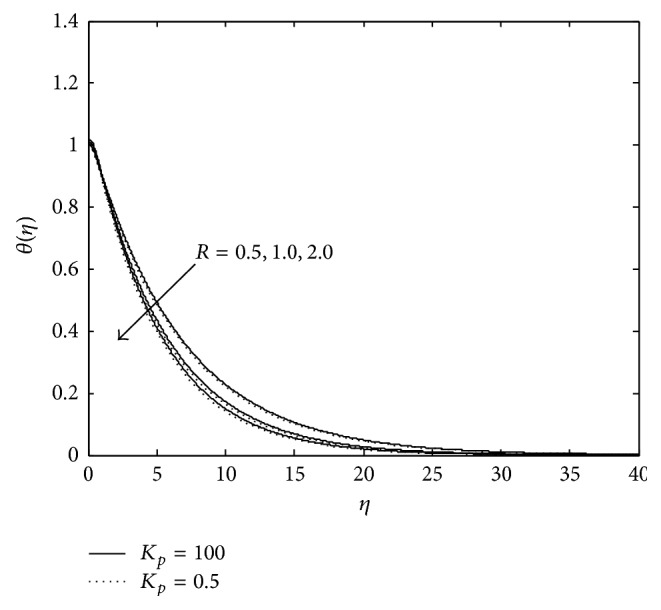
Variation of temperature: *λ* = −0.2, *M* = 3, *R*_*c*_ = 0.1, *E*_*c*_ = 0.5, and *P*_*r*_ = 0.6 (PST case).

Figure 6 exhibits the effects of *R* on the temperature field. In the absence of *K*_*p*_ (*K*_*p*_ = 100, bold lines), temperature profile reduces as *R* increases.

**Figure 7:**
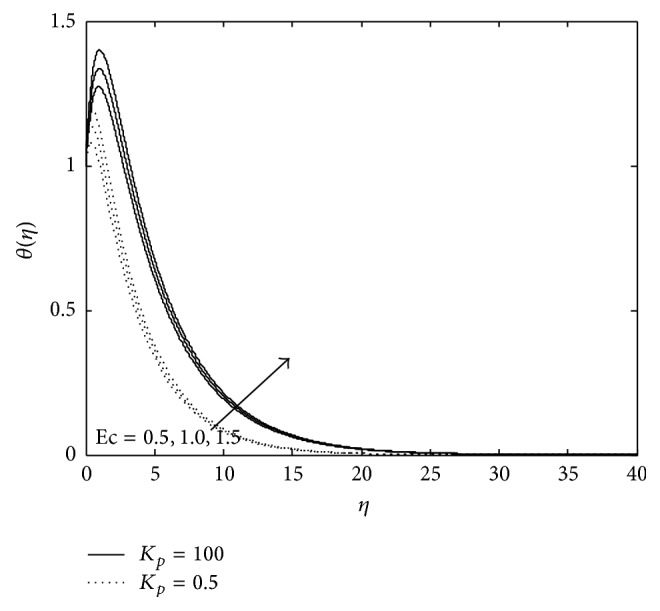
Variation of temperature.:*M* = 2, *P*_*r*_ = 0.6,  *E*_*c*_ = 0.5, *R*_*c*_ = 0.1, and *R* = 0.8 (PST case).

**Figure 8:**
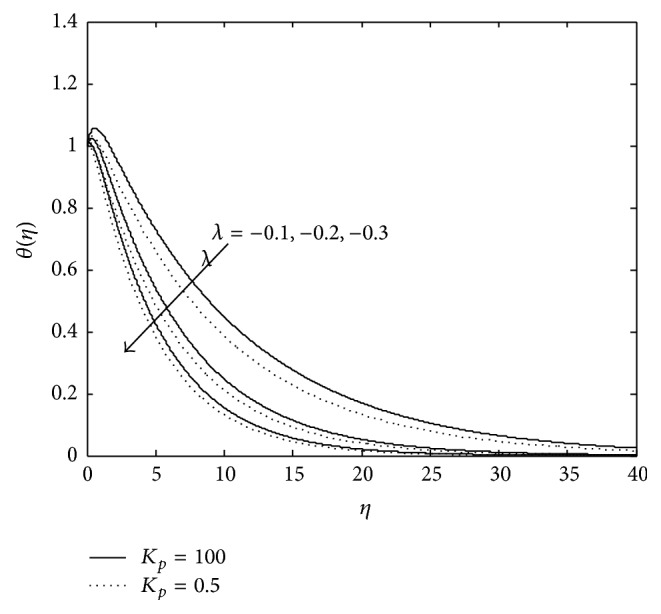
Variation of temperature: *M* = 2, *P*_*r*_ = 0.6, *E*_*c*_ = 0.5, *R*_*c*_ = 0.1, and *R* = 0.8 (PST case).

**Figure 9:**
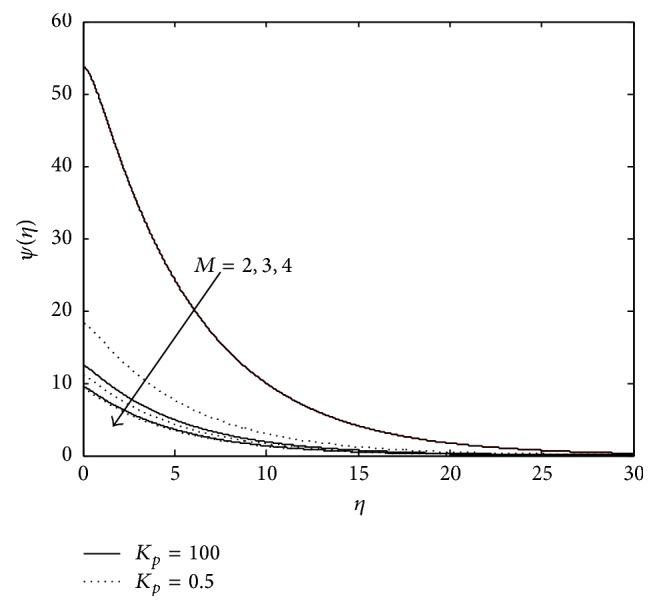
Variation of temperature: *M* = 2,3, 4,  *R* = 0.5, *R*_*c*_ = 0.1, *λ* = −0.3, *P*_*r*_ = 0.6, and *E*_*c*_ = 0.6 (PHF case).

Figures 7, 8, and 9 exhibit the effects of *R* and *E*_*c*_. An increase in *E*_*c*_ means more amount of heat energy is stored due to frictional heating, which leads to an increase in the temperature at all points and the reverse effect is observed in case of *R*. Further, it is seen that an increase in sink strength leads to a decrease in the temperature at all points (Figure 9).

**Figure 10:**
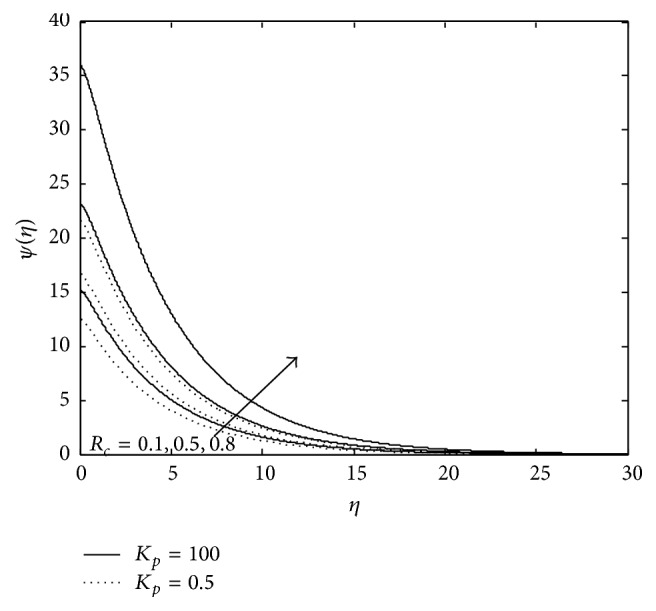
Variation of temperature: *M* = 3, *λ* = −0.3, *E*_*c*_ = 0.6, *P*_*r*_ = 0.8, and *R* = 0.6.

**Figure 11:**
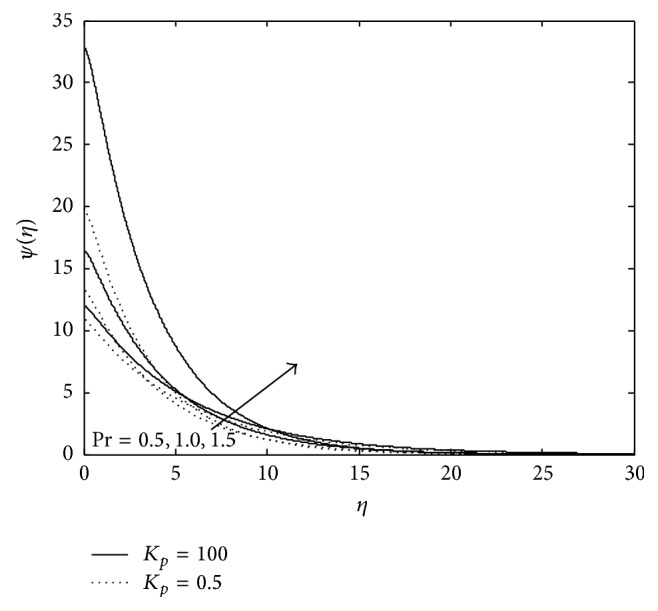
Variation of temperature: *M* = 3, *λ* = −0.3, *R*_*c*_ = 0.1, *E*_*c*_ = 0.6, and *R* = 0.5.

**Figure 12:**
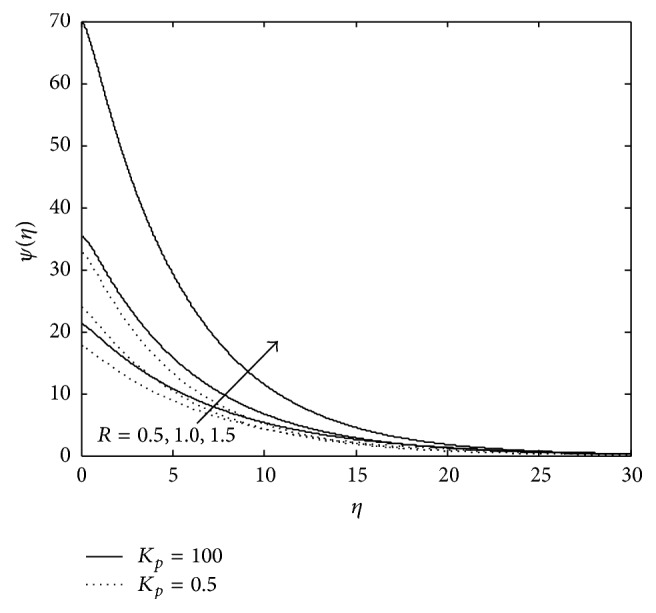
Variation of temperature: *λ* = −0.2, *R*_*c*_ = 0.1, *E*_*c*_ = 0.6, *P*_*r*_ = 0.5, and *M* = 3 (PHF Case).

**Figure 13:**
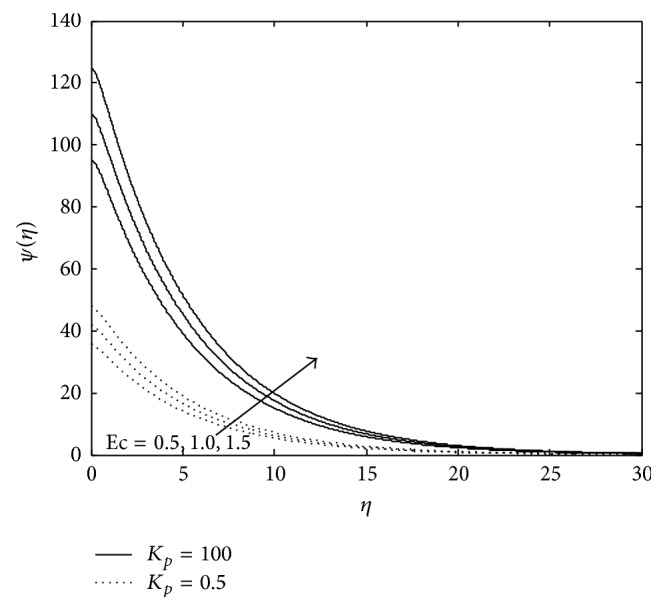
Variation of temperature: *P*_*r*_ = 0.9, *M* = 3,  *λ* = −0.2, *R*_*c*_ = 0.1, and *R* = 0.6 (PHF Case).

**Figure 14:**
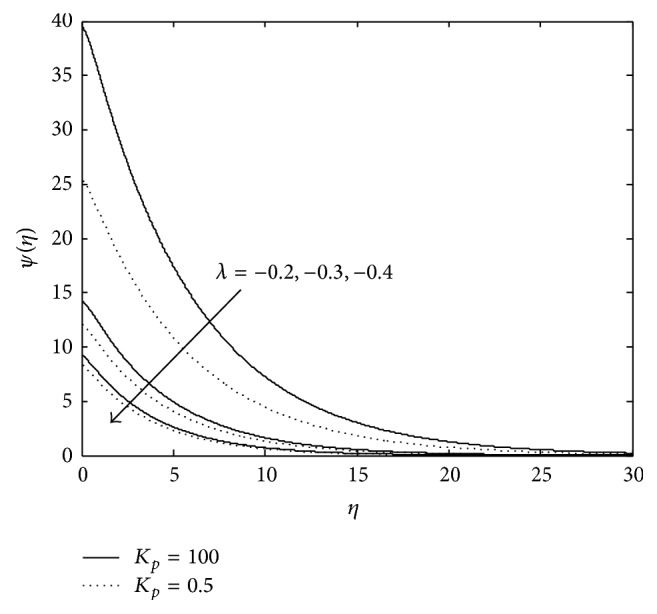
Variation of temperature: *M* = 3, *R*_*c*_ = 0.1, *P*_*r*_ = 0.6, *E*_*c*_ = 0.6, and *R* = 0.8 (PHF Case).

Figures 10, 11, 12, 13, and 14 exhibit the case of PHF. The striking feature of the temperature profiles is that the effects of the governing parameters are more pronounced in case of PHF; that is, the variation of temperature is more sensitive in the presence of heat flux. Another interesting feature of the profile is that no hike in temperature is marked in the vicinity of the plate though it was a common future in case of PST. The role of magnetic parameter, elastic parameter, Eckert number, porous matrix, and sink on temperature distribution remains the same in both PST and PHF cases except for *P*_*r*_ and *R* in both PST and PHF, but the effect of *P*_*r*_ number and *R* is with reversed effect. An increase in *P*_*r*_ leads to increase in temperature significantly within the layer (0 < *η* < 7.0), which implies that the low thermal diffusivity of fluid gives rise to higher temperature in the presence of heat flux.

**Figure 15:**
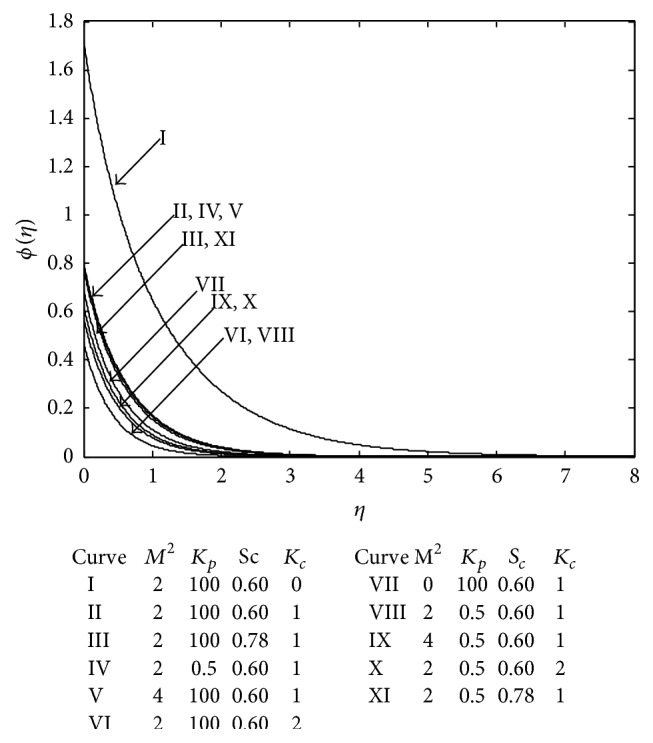
Concentration profile.

Figure 15 exhibits the concentration profile for various values of the parameters characterizing the concentration distribution. It is observed that the chemical reaction parameter reduces the concentration distribution at all points irrespective of the presence or absence of porous matrix. From the Curves VI and X it is seen that the presence of porous matrix enhances the concentration level at all points of the flow domain in the presence of chemical reaction. Now, it is further seen that the effect of magnetic field is to increase the concentration (Curve II, *M* = 0 and Curve VII, *M* = 2) but further increase in *M* has no significant effect on concentration field in the absence of porous matrix. Whereas in the presence of porous matrix (Curves IV and IX) an increase in magnetic field reduces the concentration level. Moreover, an increase in *S*_*c*_ leads to an increase in concentration in the presence of porous matrix. Thus, heavier species contribute to enhancing the level of concentration in the presence of porous matrix (Curves VIII and XI) whereas it is interesting to note that the effect of *S*_*c*_ is reverse in the absence of porous matrix (Curves II and III).

From Table 1 it is seen that an increasing magnetic parameter increases the skin friction and it is further increased by presence of porous matrix but the effect of elasticity decreases it. Thus, it is concluded that the presence of elastic elements is favourable in reducing the skin friction.

From Table 2, it is observed that the Nusselt number changes its sign from positive to negative due to presence of porous matrix that is observed from row 1 and row 2. Further, it is seen that change of sign also occurs in case of increasing the magnetic parameter, but without porous matrix. Thus, it is concluded that the effect of porous matrix is duly compensated by enhancing the magnetic field strength in the absence of porous matrix on the rate of heat transfer. Furthermore, it is seen that an increase in *P*_*r*_ and *S* (radiation parameter) leads to an increase in the Nusselt number without change of sign. Therefore, the rate of heat transfer is sensitive to the presence of porous matrix and magnetic parameter causes instability in the rate of heat transfer phenomena.

Table 3 represents the rate of mass transfer at the plate. It is interesting to observe that absence of chemical reactions contributes to the positive value, whereas presence of it gives rise to negative values.

## 8. Conclusion

- Presence of porous matrix enhances the velocity because porous matrix acts as an insulator to the vertical surface preventing energy loss due to free convection.
- The resistive force of magnetic field is overcome due to the presence of porous matrix and elasticity of the fluid and hence the velocity increases due to the presence of both.
- Further, absence of magnetic field and porous matrix leads to transitory motion of the fluid.
- Presence of elasticity also leads to an increase in the temperature at all points but the presence of magnetic field reduces it.
- The thinning of thermal boundary layer thickness is due to slow rate thermal diffusion in presence of magnetic field and porous matrix.
- The variation in temperature is more sensitive due to presence of heat flux.
- Presence of porous matrix with moderate values of magnetic parameter in case of heavier species enhances the concentration level in the presence of chemical reaction.
- For higher value of magnetic field in conjunction with porous matrix reduces the concentration level.
- Presence of elastic element is favorable in reducing the skin friction.
- The effect of porous matrix is duly compensated, enhancing the magnetic strain in the absence of porous matrix on the rate of heat transfer.
- Moreover, the rate of heat transfer in the present study is sensitive to the presence of porous matrix and magnetic parameter.
- Porous matrix enhances the rate of mass transfer, whereas increase in chemical reaction has no impact in absence of porous matrix.

## Figures and Tables

**Table 1:** Skin friction coefficient.

Sl. number	*M*	*K*_*p*_	*R*_*c*_	*τ*
1	1.5	100	0.1	1.070259
2	3	100	0.1	2.698484
3	3	0.5	0.1	3.015113
4	3	100	0.5	2.310844
5	3	100	0.8	2.109502
6	3	0.5	0.8	2.357023
7	2	100	0.1	1.654196
8	4	100	0.1	3.693975
9	2	0.5	0.1	2.132007
10	4	0.5	0.1	3.931227

**Table 2:** Nusselt number.

Sl. number	*L*	*M*	*K*_*p*_	*P*_*r*_	*E*_*c*_	*R*_*c*_	*S*	Nu
1	−0.1	2	100	0.6	0.5	0.1	0.8	0.044418
2	−0.1	2	0.5	0.6	0.5	0.1	0.8	−0.00283
3	−0.1	3	100	0.6	0.5	0.1	0.8	−0.0345
4	−0.2	2	100	0.6	0.5	0.1	0.8	−0.02321
5	−0.1	2	100	0.8	0.5	0.1	0.8	0.082496
6	−0.1	2	100	0.6	1	0.1	0.8	0.070899
7	−0.1	2	100	0.6	0.5	0.5	0.8	0.082097
8	−0.1	2	100	0.6	0.5	0.1	0.5	0.015904
9	−0.1	2	0.5	0.6	0.5	0.1	0.5	−0.01878
10	−0.1	2	0.5	0.8	0.5	0.1	0.8	0.019623
11	−0.1	3	0.5	0.6	0.5	0.1	0.8	−0.04623
12	−0.1	2	100	0.6	0	0.1	0.8	0.017938
13	−0.1	2	0.5	0.6	0	0.1	0.8	−0.02907

**Table 3:** Sherwood number.

Sl. number	*M*	*K*_*p*_	*S*_*c*_	*K*_*c*_	Sh
1	3	100	0.6	0	1.331674
2	3	100	0.6	1	−1.90499
3	3	0.5	0.6	1	−2.35153
4	4	100	0.6	1	−2.14054
5	3	100	0.78	1	−1.90499
6	3	100	0.6	2	−1.90499

## Nomenclature

*C*: Nondimensional species concentration
*C*_*∞*_: Species concentration away from the wall
*u*: Nondimensional velocity in the *x* direction
*v*: Nondimensional velocity in the *y* direction
*Q*: Volumetric rate of internal heat generation/absorption
*K*_*p*_′: Permeability of the medium
*K*: Thermal diffusivity
*R*_*c*_: Elastic parameter
*P*_*r*_: Prandtl number
*S*_*c*_: Schmidt number
*T*: Nondimensional temperature
*t*: Nondimensional time
*ρ*: Density of the fluid
*υ*: Kinematics coefficient of viscosity
*σ*^*^: Stefan-Boltzmann constant
*C*_*p*_: Specific heat
*q*_*w*_: Wall heat flux
*τ*_*w*_: Wall shear stress
*T*_*∞*_: Temperature far from sheet
*T*_*w*_: Wall temperature
*K*_*p*_: Porosity parameter
*M*: Magnetic parameter
*B*_0_: Magnetic field of uniform strength
*λ*: Heat source parameter
*T*′: Temperature of the field
*t*′: Time
*D*: Molecular diffusivity
*q*_*r*_: Radiative heat flux
*k*_1_: Absorption coefficient
*σ*: Electrical conductivity
*N*: Radiation parameter
*E*_*c*_: Eckert number
*C*_*f*_: Skin friction coefficient
*k*_0_: Dimensionless elastic parameter
*m*_*w*_: Rate of mass flux.
